# The effect of first trimester body mass index on the changes in the upper lip bite test classification before and after delivery: A prospective observational study

**DOI:** 10.3389/fmed.2022.969862

**Published:** 2022-09-14

**Authors:** Yannan Li, Yue Li, Qufei Chen, Hanli Hua, Jing Jiao, Le Zhang, Liming Chen, Shaoqiang Huang

**Affiliations:** Department of Anesthesia, Obstetrics and Gynecology Hospital, Fudan University, Shanghai, China

**Keywords:** difficult airway (DA), upper lip bite test (ULBT), labor, body mass index (BMI), parturient

## Abstract

**Background:**

The difficult airway (DA) assessment and management of pregnant woman has always brought specific challenges. The aim of this study was to investigate the effect of labor on the airway by assessing changes in the upper lip bite test (ULBT) classes and to explore its correlation with the first trimester's body mass index (BMI).

**Methods:**

According to the BMI of the first trimester, 354 full-term women were divided into low BMI group, normal BMI group and high BMI group. The ULBT class and pregnancy outcome were recorded and compared at early labor, after delivery, and 48 h after delivery.

**Results:**

The ULBT class was increased in 75(21.1%) patients after delivery. Compared to the normal BMI group, the high BMI group had a higher probability of increasing (34.8 vs. 17.5%; *P* = 0.002). The number of women with ULBT class 2–3 increased to 157, which was 1.48 times that of early labor. The number of women with ULBT class 3 increased from 4 to 16, of which 7 (53.8%) were from the high BMI group. Binary logistic regression analysis showed that first trimester's BMI was associated with a significant increase in ULBT class after delivery (adjusted odds ratio [aOR] = 2.13 [0.91–4.98], *P* = 0.02). The ULBT classes of the three groups tended to return to their initial level 48 h after delivery (*P* > 0.05).

**Conclusion:**

Labor results in an approximately one-fifth increase in ULBT class. Being overweight or obese in the first trimester is associated with an increased risk of DA during labor.

**Trial registration:**

This study was registered in the Chinese Clinical Trial Registry (http://www.chictr.org.cn) on September 26, 2020. Registration number ChiCTR2000038643.

## Introduction

Airway management in obstetric patients has always been a challenge, the incidence of failed tracheal intubation in obstetrics ranges from 0.15 to 0.6% (1), accounting for 45% of anesthesia-related maternal deaths (2). Previous studies have shown that the risk of difficult airways (DA) increases with the progression of labor (3–6), but most of these studies used the Mallampati classification as the assessment method. In recent years, the advantages of the upper lip bite test (ULBT) for predicting the risk of DA occurrence have received increasing attention. A retrospective study found that ULBT can predict intubation difficulties more conveniently and accurately than other commonly used physical examination methods (7). However, no observational study has considered changes in ULBT during labor.

The main point of this study was to use the best prediction method known so far among various types of physical examinations to confirm airway changes during labor. In addition, since obesity is a high-risk factor for maternal DA, we attempted to analyze the effect of body weight during pregnancy on the changes in ULBT during delivery.

## Materials and methods

### Study setting and participant

Ethical approval for this study (2020-6) was provided by the Ethical Committee of the Obstetrics and Gynecology Hospital of Fudan University, Shanghai, China (Chairperson Prof Congjian Xu) on 24 August 2020 and was registered in the Chinese Clinical Trial Registry (http://www.chictr.org.cn, registration number ChiCTR2000038643, 26/09/2020) before patient enrollment. Informed consent was obtained from all subjects and/or their legal guardian(s). Full-term parturients aged 18–45 years who first time gave birth in the delivery room of our hospital between October 2020 and April 2021 were recruited. The exclusion criteria were multiple gestations, previous head or neck surgery, gestational hypertension, primary psychiatric disease or a history of mental illness in the family. Women enrolled in the study who underwent cesarean section were excluded from the final analysis.

### Study protocol

Baseline information of the parturient was collected from electronic medical records and interviews and included age, height, weight, body weight in the first trimester, level of education, history of assisted reproductive technology use, and history of snoring during pregnancy. After delivery, delivery-related information was recorded, including the number of pregnancies, gestational age, total cumulative dose of oxytocin, total volume of intravenous fluids, blood loss, delivery time, newborn weight, epidural analgesia, and satisfaction with labor (satisfied, neutral, dissatisfied).

Based the body mass index (BMI) classification for Chinese adults developed by the Working Group on Obesity in China (WGOC) in 2001 (8), the mothers were divided into three groups according to their first-trimester BMI: the low BMI group (BMI < 18.5 kg/m^2^), the normal BMI group (18.5 kg/m^2^ ≤ BMI <24.0 kg/m^2^) and the high BMI group (BMI ≥ 24.0 kg/m^2^).

The main observation indicator was the change in the ULBT classification during delivery. Measurements were performed during early labor (1–3 cm dilation of the cervix), 20 min to 1 h after delivery, and 48 h after delivery. Measurement method: The parturient lay on her back with her lower jaw extended, and was asked to use her lower incisors to bite as far up the upper lip as possible. The bites were divided into three classes: Class 1, the lower incisors can reach or exceed the upper lip line; Class 2, the lower incisors can bite the upper lip below the upper lip line, and Class 3, the lower incisors cannot bite any part of the upper lip. Patients with a Class 3 ULBT result are considered at risk for a difficult tracheal intubation (7, 9).

### Sample size calculation

A pilot study involving 57 parturients (the high BMI group: the normal BMI group: the low BMI group = 11:27:9) found that the incidence of an increased ULBT class after delivery was 36.4, 18.9, and 22.2%, respectively. After introducing these incidence rates into the formula used to calculate the sample size for the comparison of multiple groups, A sample size of 321 was obtained based on α = 0.05, β = 0.10, *v* = 2, *w* = 0.17345, and λ = 9.63. The incidence of switching from vaginal delivery to cesarean section at our hospital is approximately 10–20%, and after accounting for dropouts due to other reasons, such as loss to follow-up, 430 patients were enrolled in the study.

### Statistical methods

SPSS 25.0 software was used for statistical analysis. The Shapiro–Wilk test was used to determine whether the measurement data conformed to a normal distribution. Measurement data that conformed to a normal distribution are described as $\bar{x}$ ± s, and analysis of variance (ANOVA) was used for comparisons between groups. Measurement data that did not conform to the normal distribution are expressed as the median (interquartile range). Comparisons between groups were performed using the Kruskal–Wallis nonparametric test. Count data are described as the number of cases (percentage), McNemar's test was used to compare the proportions of parturients with ULBT classes 2–3 at different time points, and the χ^2^ test was used for comparisons between groups. Two independent sample *t-*tests were used to analyze the relationship between the postpartum ULBT class increase and body weight during pregnancy. A binomial logistic regression model was used to correct potential confounding variables to analyze the effect of pregnancy weight on the ULBT. *P* < 0.05 was considered statistically significant.

## Results

**Figure 1:**
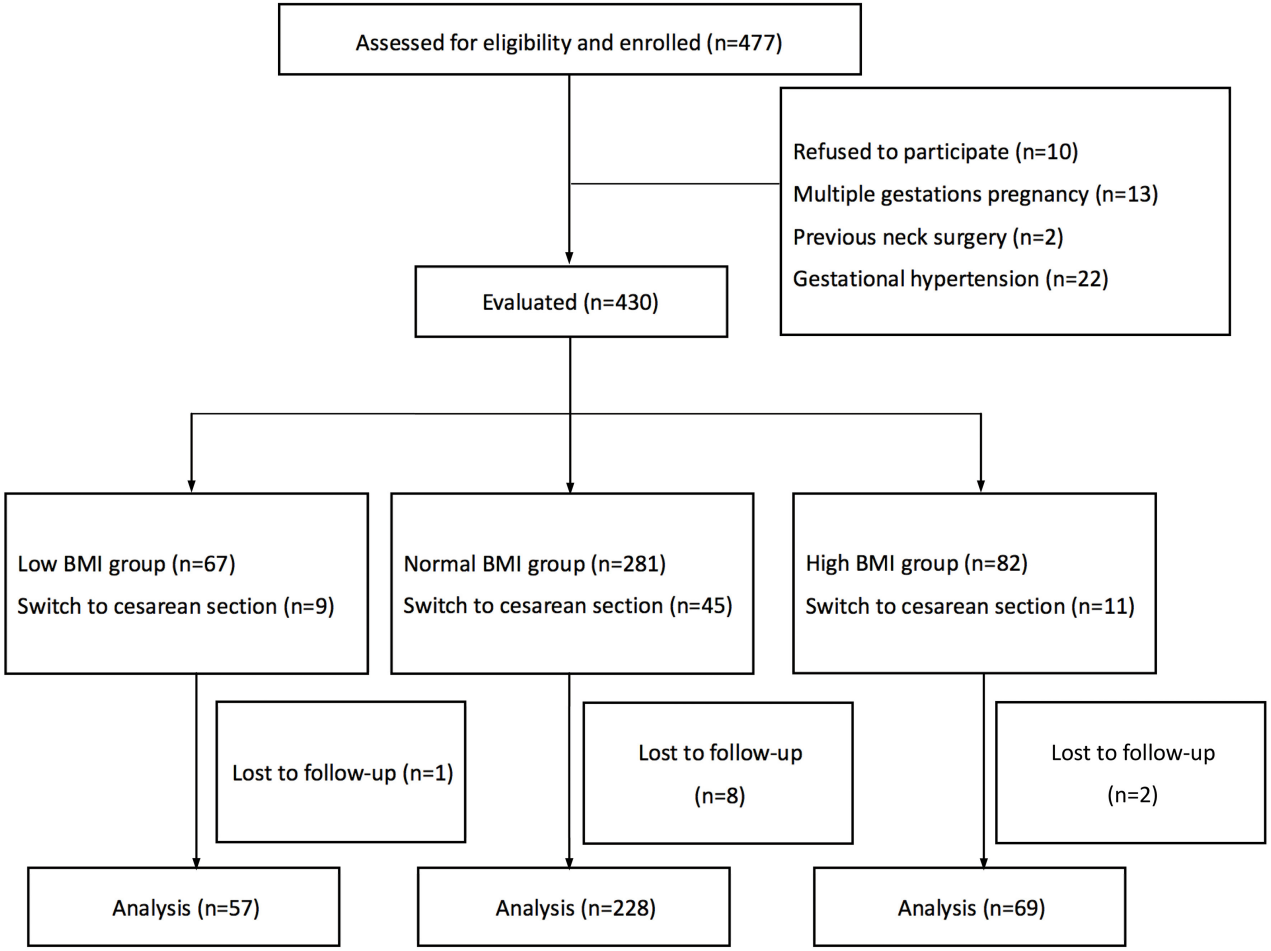
Recruitment flow chart. BMI, body mass index.

Four hundred and seventy-seven parturients were invited to participate in the study, of whom 10 declined, 13 had multiple gestation pregnancies, 2 had a history of neck surgery, and 22 had hypertension during pregnancy. We recruited 430 parturients in the study, but 65 were excluded due to a switch to cesarean section and 11 did not collect postpartum ULBT classification information due to early discharge. Finally, 354 parturients were included, with 57 in the low BMI group, 228 in the normal BMI group, and 69 in the high BMI group. The flowchart of the study is presented in Figure 1.

### Comparison of the general conditions among the three groups

There was no significant difference in age, height, number of pregnancies, gestational age, level of education, or history of assisted reproductive technology use among the three groups (*P* > 0.05). Compared with the normal BMI group, the high BMI group had a significantly higher proportion of snoring during pregnancy (*P* = 0.002) (Table 1).

**Table 1:** Baseline characteristics of included women.

	**Overall (*n =* 354)**	**Low BMI group (*n =* 57)**	**Normal BMI group (*n =* 228)**	**High BMI group (*n =* 69)**	***P***
Age; y	29.6 ± 3.5	28.7 ± 3.5	29.7 ± 3.5	30.1 ± 3.4	0.071
Height; cm	162.7 ± 4.7	163.1 ± 4.4	162.8 ± 4.9	162.1 ± 4.4	0.204
Gestational age	39.4 (38.6–40.1)	39.4 (38.5–40.1)	39.4 (38.5–39.8)	39.2 (37.9–39.6)	0.243
Weight of the first trimester; kg	56.9 ± 8.4	47.2 ± 3.3	55.8 ± 5.3	68.5 ± 6.3	<0.001
Weight at delivery; kg	69.5 ± 9.2	61.4 ± 5.4	68.4 ± 7.1	79.9 ± 8.7	0.004
Weight gain during pregnancy; kg	12.7 ± 5.2	16.4 ± 6.8	12.2 ± 4.5	11.1 ± 4.7	0.002
BMI of the first trimester; kg/m^2^	21.5 ± 3.0	17.7 ± 0.7	21.0 ± 1.5	26.2 ± 1.9	<0.001
BMI at delivery; kg/m^2^	26.3 ± 3.2	23.9 ± 2.6	25.7 ± 2.3	30.4 ± 2.8	0.003
Increase in BMI during pregnancy; kg/m^2^	4.8 ± 2.0	6.2 ± 2.6	4.6 ± 1.7	4.2 ± 1.8	0.002
**Level of education; No. (%)**					
Postgraduate and above	67 (19.0)	14 (24.6)	45 (19.7)	8 (11.6)	0.411
Undergraduate	186 (52.5)	28 (49.1)	120 (52.6)	38 (55.1)	
High school and below	101 (28.5)	15 (26.3)	63 (27.6)	23 (33.0)	
Assisted reproductive technology; No. (%)	31 (8.8%)	5 (8.7)	19 (8.3)	7 (10.1)	0.094
Snoring during pregnancy; No. (%)	175 (49.4)	20 (35.1)	105 (46.1)	50 (72.5)^*^	0.022

Values are mean (SD), median (IQR [range]) or number (proportion).

* High BMI group has a higher rate of snoring during pregnancy (P = 0.002).

### Comparison of delivery outcomes among the three groups

Compared with the normal BMI group, the high BMI group had higher oxytocin dosage (*P* = 0.049), rehydration volume (*P* < 0.001), and blood loss (*P* = 0.008). There were no significant differences in the duration of labor, newborn weight, labor analgesia use, or satisfaction with labor among the three groups (*P* > 0.05) (Table 2).

**Table 2:** Obstetrics data and labor management details in three groups.

	**Overall (*n =* 354)**	**Low BMI group (*n =* 57)**	**Normal BMI group (*n =* 228)**	**High BMI group (*n =* 69)**	***P***
Total oxytocin given; IU	3.1 ± 2.6	2.7 ± 2.5	3.0 ± 2.6	3.8 ± 2.4	0.042
Intravenous fluids; ml	692.7 ± 480.1	632.5 ± 439.7	644.8 ± 473.9	900.07 ± 439.7	<0.001
Blood loss; ml	218.0 ± 168.8	163.0 ± 83.0	217.0 ± 179.3	266.8 ± 172.9	<0.001
Duration of labor; min	551.4 ± 270.1	607.4 ± 311.1	534.0 ± 270.9	562.6 ± 224.2	0.169
Duration of first stage; min	477.1 ± 261.9	532.9 ± 311.3	463.2 ± 264.1	532.9 ± 311.3	0.196
Duration of second stage; min	68.3 ±42.9	69.1 ± 32.9	65.0 ± 39.0	78.8 ± 58.7	0.074
Duration of third stage; min	5.9 ± 4.2	5.4 ± 3.2	5.8 ± 4.2	6.7 ± 5.0	0.173
Birth weight; g	3275 ± 434	3204 ± 362	3265 ± 399	3396 ± 414	0.179
Epidural analgesia; No. (%)	303 (85.6)	50 (87.7)	194 (85.0)	59 (85.5)	0.061
**Satisfaction with labor; No. (%)**					
Satisfied	350 (98.8)	57 (100)	225 (98.7)	68 (98.6)	0.333
Neutral	4 (1.12)	0 (0)	3 (1.3)	1 (1.4)	
Dissatisfied	0 (0)	0 (0)	0 (0)	0 (0)	

Values are number (proportion), mean (SD) or median (IQR [range]).

### Changes in ULBT classification before and after delivery

**Figure 2:**
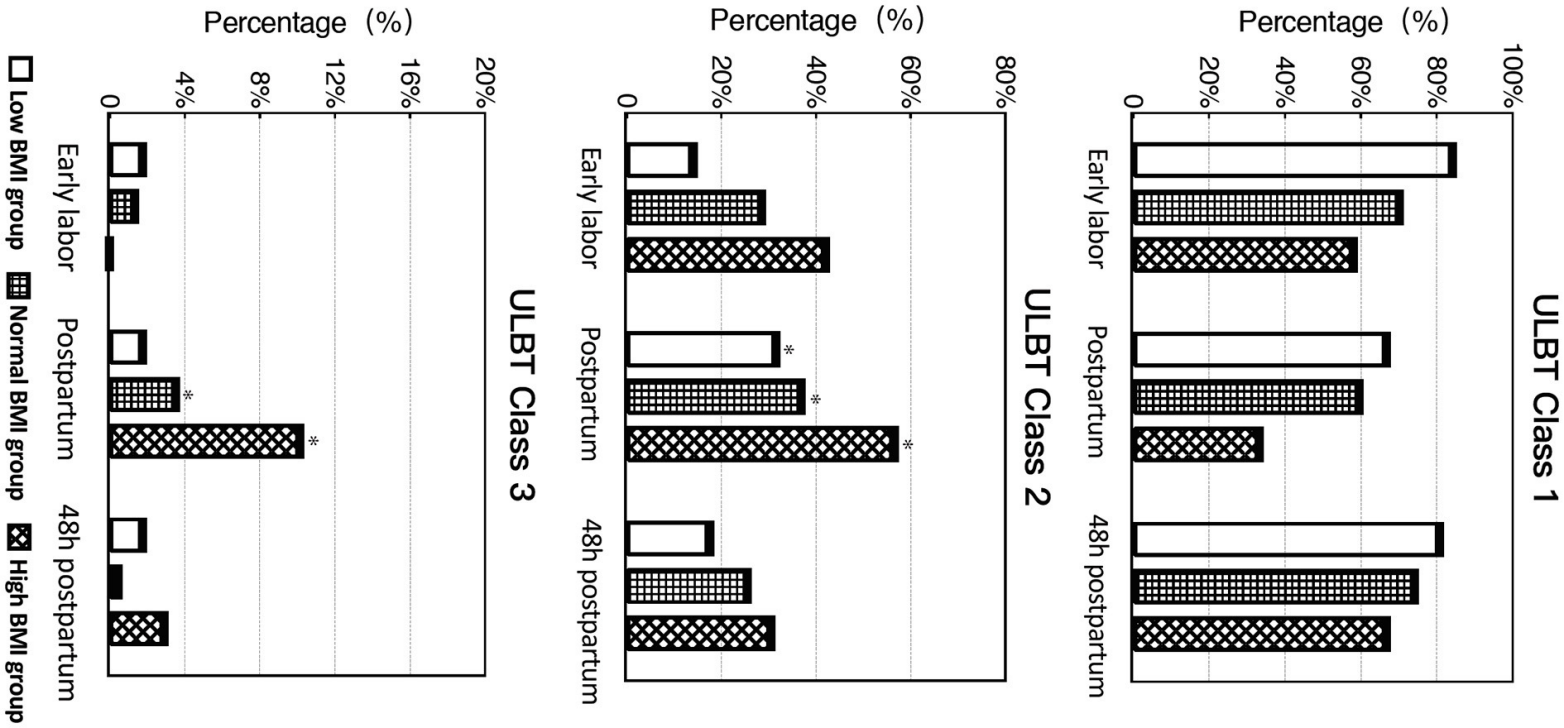
Distribution of different ULBT classifications in early labor, postpartum and 48 h postpartum. BMI, Body Mass Index; ULBT, upper lip bite test. *The proportion of ULBT level 2–3 increased significantly after delivery (*P* < 0.001) and tended to return to the level in early labor 48 h after delivery (*P* = 0.074). *The incidence of increasing in ULBT grades after delivery in the high BMI group was statistically increase compared with the normal BMI group (*P* = 0.002).

Overall, after delivery, the ULBT class increased in 75 of the 354 cases (21.1%). Among 248 parturients with ULBT class 1 before delivery, 62 (25%) increased to class 2 after delivery, and 13 (12.7%) of 102 parturients with ULBT class 2 in early labor increased to class 3 after delivery. Parturients with ULBT class 1 decreased from 248 (70.1%) in early labor to 197 (55.7%); correspondingly, women with ULBT classes 2 to 3 increased to 157, which was 1.48 times in early labor, and women with ULBT class 3 increased from 4 to 16 after delivery. The ULBT classification tended to return to the level in early labor 48 h after delivery (*P* = 0.074) (Figure 2). In terms of the three BMI groups, the ULBT classes increased by varying degrees after delivery, including an increase of 11 cases (19.3%) in the low BMI group, an increase of 40 cases (17.5%) in the normal BMI group, and an increase of 24 cases (34.8%) in the high BMI group. Compared with the normal BMI group, the incidence of increased ULBT classes in the high BMI group was statistically significant (*P* = 0.002). Among the 13 parturients whose ULBT classes increased to Class 3, 7 (53.8%) were from the high BMI group. The ULBT classifications of the three groups tended to return to those at the initial stage of labor 48h after delivery (*P* > 0.05) (Figure 2).

### The effect of body weight during pregnancy on the change in ULBT

**Figure 3:**
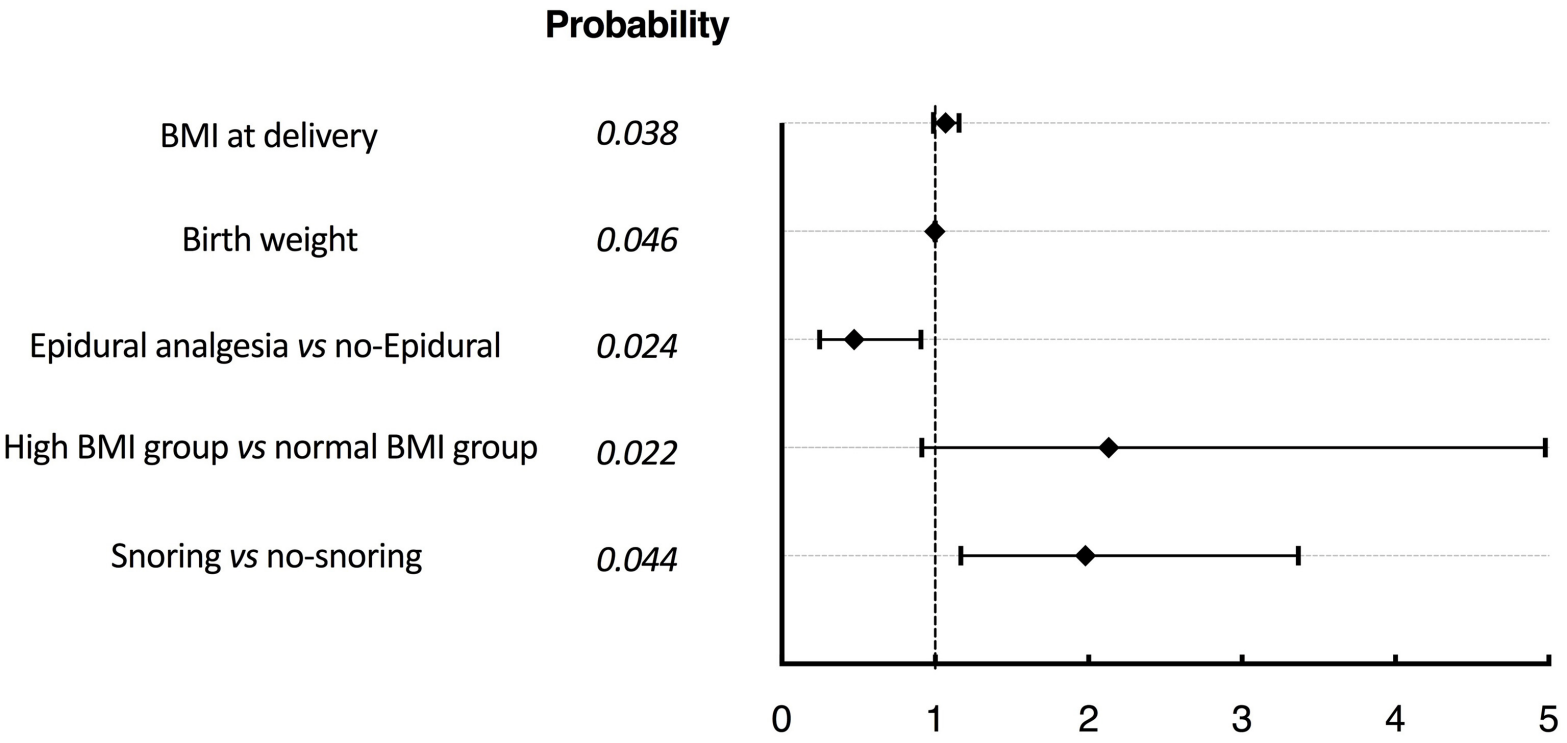
Forest plot for regression analysis of related factors affecting ULBT classification increasing after delivery. BMI, Body Mass Index; ULBT, upper lip bite test. Adjusting for these confounding factors, the binomial logistic regression analysis further confirmed that the risk of an increased ULBT class after delivery was 2.13 times higher in the high BMI group than in the normal BMI group (aOR = 2.13 [0.91–4.98], *P* = 0.02).

To further understand the effect of body weight during pregnancy on the ULBT classification change during delivery, we compared the relationship between the increase in ULBT class with the weight of the first trimester, weight at delivery, weight gain during pregnancy, BMI of the first trimester, BMI at delivery, and increase in BMI during pregnancy. We found that only BMI of the first trimester was significantly correlated with an increase in the ULBT class after delivery (*P* = 0.02). Binomial logistic regression analysis further confirmed that the risk of an increased ULBT class after delivery was 2.51 times higher in the high BMI group than in the normal BMI group (OR = 2.51 [1.35–4.58], *P* < 0.001). Other relevant risk factors included BMI at delivery, birth weight, epidural analgesia, and snoring during pregnancy. After adjusting for these confounding factors, the effect was still present (aOR = 2.13 [0.91–4.98], *P* = 0.02) (Figure 3).

## Discussion

Our study is a prospective observational study which first time explore changes in the upper lip bite test before and after labor in pregnant women and we found the risk of difficult airway is much lower compared with previous studies in this field. In fact, until now, we have not been able to determine changes in the difficult airway during labor because laryngoscopy cannot be used for a definitive Cormack-Lehane grading or endotracheal intubation in every parturient woman. Although there have been reports of an increased risk of DA during labor (3–6), the Mallampati classification is the examination method used in almost all published DA risk assessments. However, the Mallampati classification is highly subjective; the conclusions drawn by different evaluators, with different test methods, and different patient groups vary greatly among studies (10, 11). In contrast, the ULBT has high predictability and comparative variability (12), each level is clearly described and differentiated, and the judgments of different observers are relatively uniform, which allows this measure to be widely used and compared. A systematic review searched for prospective studies have found that ULBT is a useful bedside test with moderate sensitivity and PPV, and high specificity, NPV and accuracy (13). It is worth mentioning that in a review of 62 high-quality studies, Detsky et al. (7) evaluated the accuracy of various risk factors and various types of physical examinations for predicting intubation difficulties and found that the best prediction method was the ULBT, which is simple and easy to implement and has good specificity and accuracy, even for morbidly obese patients (14). This is the first study to specifically examine the changes in the risk of DA during delivery through the changes in the ULBT classification. We also used this method, which is currently regarded as the most accurate method, to verify previous evaluation results using the Mallampati classification.

We found that ULBT classes showed an increasing trend with the progression of labor. Approximately one-fifth of the parturients experienced an increase in ULBT classes after delivery, which was lower than the 66.7% increase observed in the Mallampati class, as observed by Raza and Ismail (15). The proportion of parturients with ULBT classes 2–3 increased by 48% compared with early labor and the incidence of ULBT class 3 after delivery was 4.5% on average. The ULBT class 3 is considered to be a strong indicator of intubation difficulty (9), our finding is much lower than the average Mallampati Class 4 incidence of 11.4% (5), but is similar to the DA incidence of 1 out of 19 observed in a large prospective multicenter cohort study of obstetric surgery patients by Odor et al. (16).

To facilitate comparisons with previous studies, we also evaluated the changes in the ULBT classification at 48 h postpartum. Our results showed a trend toward a decrease in ULBT class in parturients with a ULBT classes 2 or 3 during labor, which has similar conclusion with Boutonnet et al. evaluated by Mallampati class (5). The difference is that at 48 h post-labor, the ULBT classes had largely recovered to those at the beginning of labor, but the changes in Mallampati class were not fully reversed. As now we only describe a rough profile of ULBT changes and cannot make comparisons with the maternal airway status in the first trimester. Future studies should include additional time points to further clarify the changes in ULBT classes during pregnancy and delivery.

The Obstetric Anesthetists' Association and Difficult Airway Society guidelines emphasize that an increased BMI increases the risk of mask ventilation difficulties or failure (17). However, some studies have found that increased BMI itself is not a relevant predictive factor of difficult laryngoscopy (18). We found that 54.8% of women with ULBT class increased to Class 3 after delivery were overweight or obese. Regression analysis also showed that the risk of increased ULBT class after delivery in the high BMI group was 2.5 times that in the normal BMI group. Data from Odor et al. (16) showed that the proportion of obese pregnant women was higher than that in the 2013 NAP3 survey in the United Kingdom (19), and this difference was associated with the observed occurrence of intubation difficulties. A similar situation exists in China, according to the National Health Survey (20), the average BMI increased by 0.09 kg/m^2^ per year between 2010 and 2018, and the standardized average BMI level increased from 22.7 kg/m^2^ in 2004 to 24.4 kg/m^2^ in 2018. Obesity-induced pharyngeal fat deposition increases the possibility of pharyngeal wall collapse, result in higher incidence of difficult airway (21) and higher risk of rapid sequential induction intubation in obstetrics (22).

This study has several limitations. Firstly, it excluded women who switched to cesarean section, who in theory have more need for DA assessment, due to various complex reasons for cesarean delivery, such as cephalopelvic disproportion and fever during labor, etc. As the purpose of this study was to observe the impact of the entire delivery process on the ULBT classification, parturients who did not complete vaginal delivery were excluded from the study, and whether the various complex reasons for cesarean delivery increase the risk of DA remains to be further investigated. Second, in addition to the maintenance or increase of ULBT classes postpartum, this study also observed a decrease in the ULBT classes in a small number of parturients (3.4%). However, the small number of cases was not sufficient for an analysis of relevant influencing factors and future observational studies with larger sample sizes are needed. Third, the ULBT itself has some limitations. It is not suitable for edentulous patients. Although this is not a concern for most parturients, studies have found that even in edentulous patients, the upper lip catch is still a highly specific and highly sensitive indicator (23). Finally, we did not perform laryngoscopic exposure grading for the parturients. The ULBT classification is an indirect assessment method and cannot reflect the actual situation of difficult intubations. Nonetheless, its assessment is in line with our main goal, which is to use the method that is currently considered to be the most accurate predictor of DA to observe airway changes during labor, rather than to explore the essential causes of DA.

## Conclusions

The risk of DA may not be as high as we used to think, ULBT can provide bedside information that may help identify the potential DA especially when anesthesiologist have to undertake Rapid sequence induction independently. Being overweight and obesity in the first trimester are associated with an increased risk of developing DA during labor. To a certain extent, this makes us realize that the question of airway risk during labor should be revisited. Clinicians should investigate weight changes during pregnancy and perform a complete airway assessment both at admission for delivery and immediately prior to an anesthetic procedure.

## Data availability statement

The raw data supporting the conclusions of this article will be made available by the authors, without undue reservation.

## Ethics statement

Ethical approval for this study (2020-6) was provided by the Ethical Committee of the Obstetrics and Gynecology Hospital of Fudan University, Shanghai, China (Chairperson Prof. Congjian Xu) on 24 August 2020. The patients/participants provided their written informed consent to participate in this study.

## Author contributions

YaL helped design the study, conduct and administrate the study, analyze the data, and write original draft. YuL helped conduct and administrate the study, analyze the data, and write original draft. QC helped with investigation and acquisition of data. HH was responsible for investigation, statistical analysis, and interpretation. JJ helped with supervision. LZ was responsible for investigation. LC was responsible for investigation, statistical analysis, and data presentation. SH helped design the study, critically revised the manuscript for important intellectual content, and read and approved the final version of the manuscript. All authors contributed to the article and approved the submitted version.

## Conflict of interest

The authors declare that the research was conducted in the absence of any commercial or financial relationships that could be construed as a potential conflict of interest.

## Publisher's note

All claims expressed in this article are solely those of the authors and do not necessarily represent those of their affiliated organizations, or those of the publisher, the editors and the reviewers. Any product that may be evaluated in this article, or claim that may be made by its manufacturer, is not guaranteed or endorsed by the publisher.
